# The Effects of Physical Activity on Learning Behaviors in Elementary School Children: a Randomized Controlled Trial

**DOI:** 10.1007/s40688-017-0143-0

**Published:** 2017-07-17

**Authors:** Susan P. Harvey, Kate Lambourne, Jerry L. Greene, Cheryl A. Gibson, Jaehoon Lee, Joseph E. Donnelly

**Affiliations:** 1 Department of Health, Sport, and Exercise Sciences, University of Kansas, 1301 Sunnyside Avenue, Lawrence, KS 66045 USA; 2 ICF International, 9300 Lee Highway, Fairfax, VA 22031 USA; 3 Department of Internal Medicine, University of Kansas Medical Center, 3901 Rainbow Blvd., Kansas City, KS 66160 USA; 4 Educational Psychology and Leadership, Texas Tech University, 2500 Broadway Street, Lubbock, TX 79409 USA; 5 Energy Balance Laboratory, Center for Physical Activity and Weight Management, University of Kansas, 1301 Sunnyside Ave., Lawrence, KS 66045 USA

**Keywords:** Learner behaviors, Behavioral engagement, Physical activity

## Abstract

Research in education and developmental psychology indicates that behavioral engagement in learning is a critical predictor of children’s academic success. In an effort to improve academic achievement, school administrators are continually in search of methods to increase behavioral engagement. Previous research has indicated that classroom-based physical activity (PA) lessons have a positive impact on academic achievement. However, little research has been done in assessing the impact of such interventions on the behavioral engagement of students with learning behavior difficulties. This study assesses the impact of classroom-based PA on teacher-rated classroom behaviors of students with identified learning behavior difficulties. Two schools (one intervention, one control) participating in a larger, cluster-randomized trial provided scores on a teacher-administered classroom behavior scale. This scale was used to collect information on 15 characteristics identified as being essential to behavioral engagement. Participants included male and female students in second and third grade classrooms who were identified by their classroom teacher and school counselor as having difficulties with learning behaviors. Mixed linear modeling for repeated measures was used to examine the changes over time in the classroom behavior scores. The intervention group showed significant improvement over time in classroom behavior while the control group showed no change or a slight degradation over time (i.e., group × time interaction, *F*[2132] = 4.52, *p* = 0.01). Schools must meet the diverse needs of students today, including those who exhibit less than optimal learning behaviors. Combined with the evidence that PA is linked to several health and cognitive-behavior benefits, providing classroom-based PA that is incorporated within the curriculum provides common ground for all students to participate. It is a potential solution to increasing behavioral engagement, and in turn stimulating and enhancing learning.

## Background

Research in education and developmental psychology indicates that behavioral engagement in learning is a critical predictor of children’s academic success (Brophy and Good 1986; Fredricks et al. 2004; Greenwood et al. 2002). Behavioral engagement is a multifaceted concept and can be defined as a student’s active involvement in classroom tasks, including complying with classroom rules and routines, absence of disruptive behaviors (Finn 1993; Finn et al. 1995; Finn 1997), effort, persistence, attention, and concentration on tasks, and asking questions and contributing to class discussions (Finn et al. 1995; Birch and Ladd 1997; Skinner and Belmont 1993). Much research has focused on classroom-based interventions to improve behavioral engagement, as it has become a national priority for schools to demonstrate academic success.

Just as behavioral engagement is positively correlated with academic success, results of classroom-based physical activity (PA) interventions have also indicated increases in academic achievement (Centers for Disease Control and Prevention 2010). Physical activity breaks have been shown to improve some of the behavioral engagement components necessary for learning, including increased time on task (Barros et al. 2009; Gabbard and Barton 1979; Jarrett et al. 1998; Mahar et al. 2006), reduced fidgeting (Jarrett et al. 1998), and better concentration (McNaughten and Gabbard 1993).

Schools seeking the magic bullet to increase student engagement and enhance academic achievement face multiple barriers in finding a common solution. Among one of the main challenges is meeting the needs of diverse students or those who are identified as at risk for failure or dropout due to concerns in their level of engagement within the classroom. Approximately 50.1 million students attend public elementary and secondary schools, and an additional 4.9 million students attend private schools in the USA (United States Department of Education, Institute of Education Sciences, National Center for Education Statistics Common Core of Data (CCD) 2016). Among these children, it is estimated that 13–20% of them (up to 1 out of 5) experience a mental disorder in a given year (Perou et al. 2013). Mental disorders can be diagnosed and begin in childhood and include a wide range of disorders including attention-deficit/hyperactivity disorder (ADHD), Tourette syndrome, behavior disorders, mood and anxiety disorders, and autism spectrum disorders (Perou et al. 2013). Students with mental disorders can especially struggle with behavioral engagement, resulting in poor academic achievement, high rates of absenteeism, and school dropout (Finn 1997; Croninger and Lee 2001; DuPaul et al. 2004; Finn 1989). Though research is limited in this area, a few studies have indicated that students who struggle with such behaviors may benefit from engaging in PA by increasing time-on-task behaviors (Mahar et al. 2006), reducing aggressive behaviors (Gabler-Halle et al. 1993), unproductive and disruptive behaviors, and improving attention span (McKimsey and Favell 1988).

Critics of school-based PA, however, argue that classroom-based PA breaks may further disrupt the classroom environment and compete with curriculum demands (Naylor et al. 2006). Research suggesting that PA may improve behavioral engagement in the classroom challenges the notion that increasing time spent on academics is the most effective method for improving standardized test scores. Consequently, the pressure on school administrators and teachers to increase academic achievement often leads to the reduction or elimination of opportunities for PA (Mahar et al. 2006).

Recognizing that schools play an essential role in promoting student health, Congress passed the Child Nutrition and Special Supplemental Nutrition Program for Women, Infants, and Children (WIC) Reauthorization Act in 2004 (US Department of Health and Human Services 2006). This law mandates that schools with a federally funded school meal program implement wellness policies that address PA and nutrition. In 2013, the American Alliance for Health, Physical Education, Recreation, and Dance released a position statement recommending that all schools implement a comprehensive school physical activity program (CSPAP) (Elliot et al. 2013). The position statement supports recommendations by the Department of Health and Human Services and other leading public health, medical and educational organizations, and researchers that children and adolescents should be involved in moderate-to-vigorous PA at least 60 min per day (US Department of Health and Human Services 2008). The statement goes on to further emphasize the school’s role in meeting these national recommendations through high-quality physical education programs, PA during the school day, and PA before and after school by mobilizing efforts from school staff, family, and the community. However, it is recognized that even with high-quality physical education classes, students need additional opportunities for PA to meet these recommendations, and that activity breaks throughout the day are one way to help meet these guidelines, while contributing to improvements in cognitive skills, concentration, and classroom behavior.

Despite these guidelines, students spend the majority of their time in school in sedentary activities, occupying between 6 and 8 h per day in academic instruction (Donnelly and Lambourne 2011). Adding to this, research has demonstrated that children spend less than half their time in physical education classes engaged in moderate-to-vigorous PA (Levin et al. 2001). Furthermore, even with the Child Nutrition and Special Supplemental Nutrition Program for WIC Reauthorization Act, schools have for the most part focused their attention on improving the nutritional environment, all but ignoring policies to engage students and staff in PA (Kibbe et al. 2011).

While some studies have conducted observational research of how specific behavioral components, such as time on task, are affected by classroom PA breaks, to date, no known published study has used a comprehensive behavioral engagement scale to examine the impact of daily classroom-based PA on behavior with students who have previously been identified as struggling with learning behaviors. Thus, the purpose of this study was to compare learning behavior components between students who received physically active classroom-based academic lessons to those children who received lessons delivered in the traditional non-active classroom.

## Methods

### Study Design

This study was part of a larger 3-year cluster randomized, controlled trial of 17 elementary schools to determine if classroom-based PA lessons delivered by classroom teachers could enhance academic achievement significantly. The design and methods of the large cluster-randomized trial have been previously published (Donnelly et al. 2013). Briefly, 17 elementary schools were cluster randomized to intervention (A + PAAC, 9 schools) or control (8 schools) for a 3-year trial. Classroom teachers were trained to deliver academic instruction through moderate-to-vigorous PA with a target of 100 min of A + PAAC activities per week. The PA lessons were designed to be used in a variety of academic disciplines including math, language arts, geography, history, spelling, science, and health, and were directly related to the state’s core curriculum. A sample of activities are included in Table 1. The primary outcome measure was academic achievement measured by the Weschler Individual Achievement Test-III. The ancillary learning behavior study enrolled a sample of students from two schools within the same district, one delivering A + PAAC academic lessons and one control school delivering lessons in a usual manner. Levels of behavioral engagement were assessed among students identified as having difficulties with learning behaviors.

**Table 1:** Sample physically active classroom lessons

Subject	Subject topic	Physical activity
Math	Field of vision is 180°. Students stand and begin walking in place.	If 90° is 50% of the field of vision and one kangaroo jump equals 10%, how many kangaroo jumps would you perform to equal 50%? Students will do five kangaroo jumps and continue walking in place.
Spelling	Seven continents on Earth. Students stand and begin marching in place.	All continents are listed on a whiteboard. Students will spell each continent starting with the smallest to the largest. Students will hop off of both feet as they say each letter for each continent.
Language Arts	Homophones. Words will be listed on a whiteboard. Students stand begin a brisk walk in place.	As the teacher points to a word and gives it a meaning, students will do movement to show they understand, for example, peer (jog for 30 s) and pier (jump 10 times). Continue walking until next homophone is given.
Science	Force and speed. Students start with a sitting position that is easy for them to stand and sit in a safe manner.	On cue, students “slowly” raise from a sitting position to a standing position. Now, slowly lower the body back to a sitting position. Do this five times. Now raise the body to a standing position as “fast” as you can. Lower the body to the chair as fast as you can. Do this three times. Stand and start walking in place as we discuss the differences in force and speed.

With all physical activity, safety is discussed with the students. The curriculum content and the physical activity will vary among grade levels. The above sample activities are directly related to the state’s core curriculum and serve as examples of how PA can easily be part of the classroom learning process.

### Participants

Participants included male and female students in second and third grade general education classrooms at two urban public schools in northeast Kansas. Racial/ethnicity information was not collected; however, both school buildings report white students as their primary demographic makeup (>70%). Parents of students who were identified by their classroom teacher and school counselor as having difficulties with learning behaviors or were on individual education plans (IEPs) targeting behavioral goals were approached for this study. Students returning signed parental informed consent forms were assessed on 15 outcome variables related to behavioral engagement. Table 2 provides a summary of the sample characteristics. This study was approved by the institutional review boards at the University of Kansas and the school district.

**Table 2:** Sample characteristics

	Intervention	Control	Total
Male			
Second grade	17	5	22
Third grade	4	5	9
Female			
Second grade	11	10	21
Third grade	11	5	16
Total	43	25	68

### Instruments

A learning behavior scale measuring general learner outcomes (GLOs) and developed by the Hawaii Department of Education (Education HD of General Learner Outcome Scale 2016) was utilized for this study. The behavior scale was adopted by the district for use by classroom teachers and had been utilized for several years. The scale collected information on 15 observable characteristics identified in the literature as being essential to behavioral engagement, such as effort, work habits, and cooperation skills, and are evaluated separately from academic performance. Students were rated on behaviors using four criteria: (1) “E,” excels: consistently goes beyond learner behavior expectations. Evidence of most recent work demonstrates that the learner behavior goals are fully and consistently met; (2) “S,” successfully meets: meets learner behavior expectations. Evidence of most recent work demonstrates that the learner behavior goals are fully and consistently met; (3) “M,” making progress: partially meets learner behavior expectations. Evidence of most recent work demonstrates that more than half the learner behavior goals are fully and consistently met; and (4) “T,” targeted: targeted for growth in order to meet learner behavior expectations. Evidence of most recent work demonstrates that only a few of the learner behavior goals are met or partially met. Though the scale had been adopted for use from a separate district, reliability and validity properties of the scale are unknown. Table 3 shows the scale used for this study.

**Table 3:** Learner Behaviors Scale

Learner behaviors	Trimester 1, September (baseline)	Trimester 2, December	Trimester 3, March
Shows acceptance to other ideas			
Respects others (teachers, substitutes, paras, student teachers, peers, etc.)			
Actively listens			
Responds appropriately to feedback			
Uses materials purposefully and respectfully			
Follows directions			
Uses organizational strategies and organizes classroom materials/personal belongings			
Uses time efficiently and constructively			
Strives to produce quality work			
Completes tasks on time (classroom/homework)			
Manages transitions and changes in routine			
Exercises self-control			
Accepts responsibility for behavior			
Works quietly and stays on task			
Uses cooperation skills (whole group, small group, partners)			
Key for learner behaviors			
E = excels: consistently goes beyond learner behavior expectations. Evidence of most recent work demonstrates the learner behavior goals are fully and consistently met S = successfully meets: meets learner behavior expectations. Evidence of most recent work demonstrates the learner behavior goals are fully and consistently met M = making progress: partially meets learner behavior expectations. Evidence of most recent work demonstrates more than half the learner behavior goals are fully and consistently met T = targeted for growth in order to meet learner behavior expectations. Evidence of most recent work demonstrates only a few of the learning goals are met or partially met			

### Procedures

In accordance with district policies, each school year teachers and counselors worked closely together to identify and monitor students with learning behavior difficulties. Counselors trained teachers in how to complete the scale, and prior to completion, teachers and the school counselor would meet to again discuss completion of the scale to ensure accuracy. Once the scale was completed on each identified student, the teacher and counselor would meet again to debrief on the results of the scale for each student and identify interventions to increase engagement of students who were particularly struggling. Accuracy in completion of the scale was critical, as the results of the scale followed each identified student throughout their school career in order to monitor progress, address concerns, and develop interventions to attend to problematic behaviors. The scale was completed on each identified student at three time points across the school year, usually near the beginning of the trimester (September, December, March).

### Data Analysis

The participants’ demographics and classroom behavior scores were summarized by descriptive statistics and bivariate tests. Then, mixed linear modeling for repeated measures was used to examine the changes over time in the scores for the intervention and control groups. Specifically, mixed models estimated the effects of time, group, and time-by-group interaction accounting for the participants’ gender (Hox 1995; Maas and Hox 2004; Maas and Hox 2005). Statistical significance was determined at the 0.05 alpha level, and all analyses were conducted using SAS 9.4 (SAS Institute 2002-2012).

## Results

Students in second and third grade classrooms identified by their teacher and school counselor as having learning behavior difficulties were tracked with the behavioral engagement scale at three time points over the course of the school year. Within the intervention school, 21 male and 22 female (*n* = 43) students were measured. In the comparison school, 10 male and 15 female (*n* = 25) students were assessed. Distributions of grade (*χ* ^2^ [1] = 0.18, *p* = 0.67, Cramer’s *V* = 0.05) and gender (*χ* ^2^ [1] = 0.50, *p* = 0.48, *V* = 0.09) did not differ between groups.

**Fig. 1:**
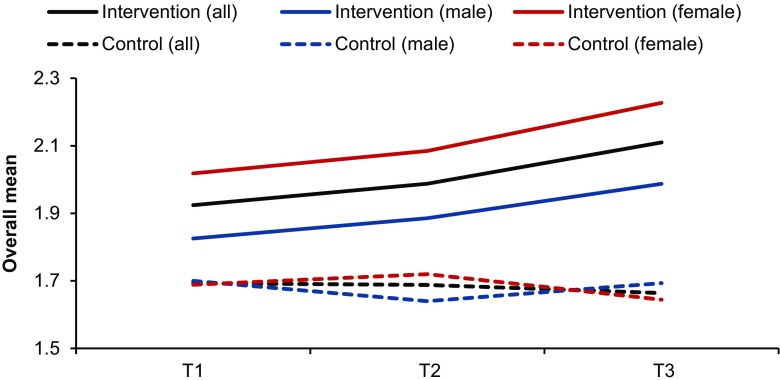
Intervention vs. control mean learner behavior scores across time

Multivariate assumptions were checked prior to analyses. Standardized skewness scores and the Shapiro-Wilk test results confirmed normality of the overall classroom behavior score at each time point within each group. The Levene’s test results also indicated homogenous variances of the overall score between groups. Table 4 provides results for the intervention group for each of the behavioral engagement components across time. Table 5 shows the mean scores for both groups across time. Results of the mixed linear modeling for repeated measures indicated that the intervention group of students receiving the classroom-based physically active lessons showed significant improvements over time in the overall behavior engagement score, i.e., slow then steep increases in the score (95% CI for change from T1 to T2 = [0.00, 0.13], Cohen’s *d* = 0.43, 95% CI for change from T2 to T3 = [0.06, 0.19], *d* = 0.81; *F*(2, 84) = 17.29, *p* < 0.0001). Results for the control group showed no change or a slight degradation over time (95% CI for change from T1 to T2 = [−0.09, 0.08], *d* = 0.03, 95% CI for change from T2 to T3 = [−0.11, 0.06], *d* = 0.16; *F*[2, 48] = 0.26, *p* = 0.77). This pattern of changes was also confirmed by a significant group-by-time interaction in a subsequent mixed modeling analysis (*F*(2, 132) = 4.52, *p* < 0.05). Figure 1 illustrates this group-by-time interaction.

**Table 4:** Intervention student mean scores and time effect

	T1		T2		T3			
	*M*	*SD*	*M*	*SD*	*M*	*SD*	*F*	*p*
Male (*n* = 21)								
Shows acceptance of other ideas	1.95	0.59	2.00	0.45	2.05	0.50	1.54	0.227
Respects others	1.90	0.70	2.14	0.73	2.14	0.73	4.95	0.012
Actively listens	1.90	0.62	2.10	0.70	2.05	0.74	3.66	0.035
Responds appropriately to feedback	1.76	0.62	2.00	0.63	1.95	0.67	3.33	0.046
Uses materials purposefully and respectfully	2.10	0.54	2.05	0.74	2.14	0.57	1.00	0.377
Follows directions	1.67	0.58	1.71	0.78	1.86	0.65	1.48	0.240
Uses organizational strategies	2.05	0.59	1.95	0.80	2.05	0.67	0.49	0.618
Uses time efficiently	1.57	0.60	1.81	0.75	2.00	0.63	7.18	0.002
Strives to produce quality work	1.81	0.51	1.81	0.51	2.00	0.71	1.65	0.205
Completes tasks on time	1.86	0.36	2.00	0.63	2.10	0.54	1.99	0.150
Manages transitions and changes in routine	1.86	0.48	1.86	0.65	2.05	0.38	2.91	0.066
Exercises self-control	1.57	0.68	1.52	0.75	1.62	0.67	0.49	0.618
Accepts responsibility for behavior	1.90	0.70	1.90	0.70	2.05	0.59	0.40	0.104
Works quietly and stays on task	1.67	0.58	1.62	0.67	1.71	0.56	0.36	0.697
Uses cooperation skills	1.81	0.51	1.81	0.60	2.05	0.74	4.10	0.024
Mean score	1.83	0.39	1.89	0.47	1.99	0.46	6.90	0.003
Female (*n* = 22**)**								
Shows acceptance of other ideas	2.09	0.29	2.14	0.35	2.23	0.43	2.49	0.095
Respects others	2.09	0.29	2.18	0.39	2.27	0.46	3.32	0.046
Actively listens	2.00	0.44	2.09	0.68	2.09	0.53	0.56	0.575
Responds appropriately to feedback	1.95	0.21	2.05	0.38	2.05	0.38	2.10	0.135
Uses materials purposefully and respectfully	2.00	0.44	2.05	0.72	2.27	0.63	4.49	0.017
Follows directions	2.00	0.31	2.27	0.55	2.36	0.58	8.81	0.001
Uses organizational strategies	2.09	0.43	2.23	0.61	2.23	0.69	0.90	0.416
Uses time efficiently	2.05	0.38	2.00	0.62	2.23	0.61	3.32	0.046
Strives to produce quality work	2.00	0.31	1.95	0.49	2.27	0.46	6.79	0.003
Completes tasks on time	1.91	0.29	2.09	0.68	2.14	0.64	2.49	0.095
Manages transitions and changes in routine	2.09	0.29	2.05	0.49	2.32	0.57	4.49	0.017
Exercises self-control	2.00	0.31	2.14	0.47	2.32	0.57	4.25	0.021
Accepts responsibility for behavior	2.09	0.29	2.09	0.29	2.18	0.39	2.10	0.135
Works quietly and stays on task	1.91	0.53	1.86	0.56	2.14	0.56	6.45	0.004
Uses cooperation skills	2.00	0.00	2.09	0.29	2.32	0.48	7.12	0.002
Mean score	2.02	0.24	2.08	0.36	2.23	0.41	10.15	0.001
Combined (*n* = 43)								
Shows acceptance of other ideas	2.02	0.46	2.07	0.40	2.14	0.47	4.07	0.021
Respects others	2.00	0.53	2.16	0.57	2.21	0.60	7.75	0.001
Actively listens	1.95	0.53	2.09	0.68	2.07	0.63	2.95	0.058
Responds appropriately to feedback	1.86	0.47	2.02	0.51	2.00	0.53	5.25	0.007
Uses materials purposefully and respectfully	2.05	0.49	2.05	0.72	2.21	0.60	4.85	0.010
Follows directions	1.84	0.48	2.00	0.72	2.12	0.66	7.36	0.001
Uses organizational strategies	2.07	0.51	2.09	0.72	2.14	0.68	0.38	0.683
Uses time efficiently	1.81	0.55	1.91	0.68	2.12	0.63	8.71	0.001
Strives to produce quality work	1.91	0.43	1.88	0.50	2.14	0.60	7.03	0.002
Completes tasks on time	1.88	0.32	2.05	0.65	2.12	0.59	4.50	0.014
Manages transitions and changes in routine	1.98	0.41	1.95	0.58	2.19	0.50	7.48	0.001
Exercises self-control	1.79	0.56	1.84	0.69	1.98	0.71	3.43	0.037
Accepts responsibility for behavior	2.00	0.53	2.00	0.53	2.12	0.50	4.51	0.014
Works quietly and stays on task	1.79	0.56	1.74	0.62	1.93	0.59	3.97	0.023
Uses cooperation skills	1.91	0.37	1.95	0.49	2.19	0.63	10.90	0.001
Mean score	1.92	0.33	1.99	0.42	2.11	0.45	17.29	0.001

**Table 5:** Intervention vs. control mean scores

	Intervention						Control					
	T1		T2		T3		T1		T2		T3	
	*M*	*SD*	*M*	*SD*	*M*	*S*D	*M*	*SD*	*M*	*SD*	*M*	*SD*
Learner behavior	Male *n* = 21						Male *n* = 10					
Shows acceptance of other ideas	1.95	0.59	2.00	0.45	2.05	0.50	1.60	0.84	1.60	0.52	1.90	0.74
Respects others	1.90	0.70	2.14	0.73	2.14	0.73	1.80	0.63	1.70	0.67	2.20	0.63
Actively listens	1.90	0.62	2.10	0.70	2.05	0.74	1.70	0.67	1.70	0.67	1.80	0.42
Responds appropriately to feedback	1.76	0.62	2.00	0.63	1.95	0.67	1.80	0.42	1.70	0.48	1.70	0.48
Uses materials purposefully and respectfully	2.10	0.54	2.05	0.74	2.14	0.57	1.60	0.70	1.40	0.70	1.70	0.67
Follows directions	1.67	0.58	1.71	0.78	1.86	0.65	2.00	0.47	1.80	0.63	1.50	0.53
Uses organizational strategies	2.05	0.59	1.95	0.80	2.05	0.67	1.70	0.48	1.80	0.42	1.90	0.57
Uses time efficiently	1.57	0.60	1.81	0.75	2.00	0.63	1.70	0.48	1.60	0.52	1.50	0.53
Strives to produce quality work	1.81	0.51	1.81	0.51	2.00	0.71	1.40	0.84	1.50	0.71	1.40	0.70
Completes tasks on time	1.86	0.36	2.00	0.63	2.10	0.54	1.60	0.70	1.30	0.82	1.50	0.53
Manages transitions and changes in routine	1.86	0.48	1.86	0.65	2.05	0.38	1.60	0.70	1.60	0.70	1.40	0.52
Exercises self-control	1.57	0.68	1.52	0.75	1.62	0.67	1.70	0.48	1.70	0.48	1.70	0.67
Accepts responsibility for behavior	1.90	0.70	1.90	0.70	2.05	0.59	1.80	0.42	1.70	0.48	1.80	0.42
Works quietly and stays on task	1.67	0.58	1.62	0.67	1.71	0.56	1.70	0.67	1.90	0.32	1.90	0.32
Uses cooperation skills	1.81	0.51	1.81	0.60	2.05	0.74	1.80	0.63	1.60	0.70	1.50	0.53
Overall mean	1.83	0.39	1.89	0.47	1.99	0.46	1.70	0.32	1.64	0.32	1.69	0.27
	Female *n* = 22						Female *n* = 15					
Shows acceptance of other ideas	2.09	0.29	2.14	0.35	2.23	0.43	1.73	0.70	1.80	0.77	1.73	0.70
Respects others	2.09	0.29	2.18	0.39	2.27	0.46	1.80	0.68	1.80	0.68	1.60	0.51
Actively listens	2.00	0.44	2.09	0.68	2.09	0.53	1.67	0.49	1.73	0.46	1.67	0.52
Responds appropriately to feedback	1.95	0.21	2.05	0.38	2.05	0.38	1.60	0.74	1.80	0.41	1.60	0.51
Uses materials purposefully and respectfully	2.00	0.44	2.05	0.72	2.27	0.63	1.67	0.62	1.73	0.70	1.53	0.52
Follows directions	2.00	0.31	2.27	0.55	2.36	0.58	1.73	0.80	1.87	0.52	1.60	0.51
Uses organizational strategies	2.09	0.43	2.23	0.61	2.23	0.69	1.73	0.46	1.67	0.49	1.67	0.49
Uses time efficiently	2.05	0.38	2.00	0.62	2.23	0.61	1.53	0.52	1.73	0.46	1.53	0.52
Strives to produce quality work	2.00	0.31	1.95	0.49	2.27	0.46	1.53	0.64	1.67	0.49	1.67	0.49
Completes tasks on time	1.91	0.29	2.09	0.68	2.14	0.64	1.73	0.59	1.60	0.63	1.60	0.51
Manages transitions and changes in routine	2.09	0.29	2.05	0.49	2.32	0.57	1.87	0.74	1.73	0.70	1.60	0.63
Exercises self-control	2.00	0.31	2.14	0.47	2.32	0.57	1.80	0.56	1.93	0.46	1.80	0.41
Accepts responsibility for behavior	2.09	0.29	2.09	0.29	2.18	0.39	1.80	0.77	1.67	0.72	1.87	0.64
Works quietly and stays on task	1.91	0.53	1.86	0.56	2.14	0.56	1.53	0.52	1.47	0.52	1.53	0.52
Uses cooperation skills	2.00	0.00	2.09	0.29	2.32	0.48	1.60	0.74	1.60	0.63	1.67	0.49
Mean score	2.02	0.24	2.08	0.36	2.23	0.41	1.69	0.35	1.72	0.30	1.64	0.27
	Total *n* = 43						Total *n*​ ﻿﻿= 25					
Shows acceptance of other ideas	2.02	0.46	2.07	0.40	2.14	0.47	1.68	0.75	1.72	0.68	1.80	0.71
Respects others	2.00	0.53	2.16	0.57	2.21	0.60	1.80	0.65	1.76	0.66	1.84	0.62
Actively listens	1.95	0.53	2.09	0.68	2.07	0.63	1.68	0.56	1.72	0.54	1.72	0.46
Responds appropriately to feedback	1.86	0.47	2.02	0.51	2.00	0.53	1.68	0.63	1.76	0.44	1.64	0.49
Uses materials purposefully and respectfully	2.05	0.49	2.05	0.72	2.21	0.60	1.64	0.64	1.60	0.71	1.60	0.58
Follows directions	1.84	0.48	2.00	0.72	2.12	0.66	1.84	0.69	1.84	0.55	1.56	0.51
Uses organizational strategies	2.07	0.51	2.09	0.72	2.14	0.68	1.72	0.46	1.72	0.46	1.76	0.52
Uses time efficiently	1.81	0.55	1.91	0.68	2.12	0.63	1.60	0.50	1.68	0.48	1.52	0.51
Strives to produce quality work	1.91	0.43	1.88	0.50	2.14	0.60	1.48	0.71	1.60	0.58	1.56	0.58
Completes tasks on time	1.88	0.32	2.05	0.65	2.12	0.59	1.68	0.63	1.48	0.71	1.56	0.51
Manages transitions and changes in routine	1.98	0.41	1.95	0.58	2.19	0.50	1.76	0.72	1.68	0.69	1.52	0.59
Exercises self-control	1.79	0.56	1.84	0.69	1.98	0.71	1.76	0.52	1.84	0.47	1.76	0.52
Accepts responsibility for behavior	2.00	0.53	2.00	0.53	2.12	0.50	1.80	0.65	1.68	0.63	1.84	0.55
Works quietly and stays on task	1.79	0.56	1.74	0.62	1.93	0.59	1.60	0.58	1.64	0.49	1.68	0.48
Uses cooperation skills	1.91	0.37	1.95	0.49	2.19	0.63	1.68	0.69	1.60	0.65	1.60	0.50
Mean score	1.92	0.33	1.99	0.42	2.11	0.45	1.69	0.33	1.69	0.30	1.66	0.27

## Discussion

School teachers and administrators are increasingly concerned with providing evidence of academic achievement through standardized test scores. In order to increase these scores, schools often find themselves restricting or eliminating time spent in PA in order to dedicate more time spent in the classroom. These actions, however, do not support a healthy school environment as mandated by the Child Nutrition and Special Supplemental Program for Women, Infants, and Children in which policies addressing PA be implemented, enforced, and evaluated.

Additionally, school districts are struggling to meet the diverse needs of students today. Homelessness, hunger, mood disorders, and ADHD are just a few examples of how a student’s learning outcomes may be influenced by other stressors. Students who face significant hardships outside of school or have mental challenges often struggle with school and classroom engagement.

Because school administrators recognize the importance of behavioral engagement and its linkages to academic achievement, they have sought interventions for those students who are struggling. However, well-intentioned interventions often aim to address intrapersonal and interpersonal factors of struggling students (e.g., academic, social, and personal problems) in order to address behavioral engagement (Lehr et al. 2003). As a result, these individual-level interventions may isolate a student by pulling them from their classroom or singling them out during classroom activities. It is important to note that behavioral engagement, in part, includes social-emotional components related to teacher-to-student relationships (Cadima et al. 2015; Pianta and Stuhlman 2004) and student-to-student relationships (Downer 2007). Interventions that focus only on intrapersonal and interpersonal factors may therefore compromise relationships with teachers and peers that are essential to fostering social-emotional engagement and supporting positive learner behaviors.

Results of studies that have increased the amount of PA through classroom-based PA breaks have for the most part shown positive results in both behavioral engagement and academic achievement with improvements in time on task (Mahar et al. 2006), classroom behavior (Gabler-Halle et al. 1993; McKimsey and Favell 1988), attention span (McKimsey and Favell 1988), and test scores (Centers for Disease Control and Prevention 2010). Though a few studies have noted no differences between classroom-based PA and academic achievement, it is important to note that to date, no study has shown there to be any detrimental effects of classroom-based PA on behavior or academic achievement (Centers for Disease Control and Prevention 2010).

The impact that increased levels and minutes of PA can have on students who especially struggle with learning behaviors is a possible intervention that is inclusive for all students in the classroom and does not compete for instructional time. Classroom-based PA provides common ground for students to participate with each other and their teacher in an active learning environment.

The results of the present study clearly indicated that participation in classroom-based PA for students who struggle with learning behaviors enhanced several domains of behavioral engagement. Students participating in physically active academic lessons displayed significant improvements in teacher-rated classroom behaviors, while students in the traditional non-active classroom exhibited little or slight degradation in the same behaviors. It should be noted that intrapersonal skills (e.g., exercises self-control, use of time, attention to task) and social-emotional skills (e.g., acceptance of other ideas, respect for others, cooperation skills) exhibited significant improvements over the course of the year for the physically active group. Classrooms integrating PA in short increments throughout the school day provide a feasible way to engage both teachers and students and meet the requirements set forth by the Child Nutrition and Special Supplemental Nutrition Program for Women, Infants, and Children Reauthorization Act.

### Limitations

There are limitations to this study, most notably in the instrument and methods used in obtaining the data. While the learning behavior instrument had been adopted from a different school district who also used the scale, to our knowledge, it has not undergone any validity or reliability testing. Secondly, the data were collected by classroom teachers and is subjective in nature and no inter-rater reliability testing was conducted. However, since the district utilizes the learning behavior scale to monitor students through their academic career, extra care is taken by teachers and counselors to ensure precision and accuracy in measuring students and identifying appropriate interventions. Third, we did not collect academic data for these students; thus, while components of behavioral engagement significantly improved in the intervention students, we do not know if this improvement also influenced academic achievement. Fourth, due to the sensitivity of the subject matter and to comply with district request, we did not collect specific information on student learning or mental disorders. Finally, because only one school district was identified in utilizing such a comprehensive measure for determining general learner outcomes among students identified with learning behavior difficulties, the sample size for this study is small.

## Conclusions

Schools face a multitude of barriers today in addressing the academic challenges of students, as well as attending to physical, emotional, and mental health challenges that many students face. Of particular concern are those students who are identified with difficulties in learning behaviors. These students are at increased risk for poor academic performance despite the school administration’s best efforts to address problem behaviors.

Evidence clearly indicates that moderate-to-vigorous PA can have an immense impact on not only physical health but also mental health and cognition. In accordance with the Child Nutrition and Special Supplemental Nutrition Program for WIC Reauthorization Act, all school districts must have policies in place that address PA. While schools maintain that demonstration of academic achievement is a priority, ensuring the health and wellness of students is also of critical importance. Though students may receive PA through physical education classes, more programs to facilitate and encourage moderate-to-vigorous PA throughout the school day must also be in place.

Combined with evidence that PA is linked to so many health and cognitive-behavioral benefits, classroom-based PA provides common ground for all students to participate without reducing time allocated for classroom instruction. Physically active lessons enhance students’ behavioral engagement and, in turn, stimulate and enhance learning. Future research should further examine how physically active classroom lessons impact both learner behavior outcomes and academic achievement utilizing similar scales on students with identified behavioral engagement problems, including those students with learning and mental disorders.
